# Effect of pirfenidone protecting against cigarette smoke extract induced apoptosis

**DOI:** 10.18332/tid/146169

**Published:** 2022-03-01

**Authors:** Yiming Ma, Xiangming Liu, Lijuan Luo, Herui Li, Zihang Zeng, Yan Chen

**Affiliations:** 1 Department of Respiratory and Critical Care Medicine, The Second Xiangya Hospital, Central South University, Changsha, China

**Keywords:** chronic obstructive pulmonary disease, emphysema, pirfenidone, apoptosis, treatment

## Abstract

**INTRODUCTION:**

Apoptosis of lung structural cells is a significant upstream event involved in COPD pathogenesis. This study was designed to explore whether pirfenidone (PFD) was able to attenuate apoptosis induced by cigarette smoke extract (CSE).

**METHODS:**

A method of intraperitoneal CSE injection to BALB/C mice was used to establish emphysema mouse model. Terminal deoxynucleotidyl transferase dUTPnick end labeling (TUNEL) assay was applied to evaluate apoptotic cell ratio in mouse lung tissue. The cell viability of HBECs exposed to different concentrations of PFD was measured by Cell Counting Kit-8 (CCK-8) assay. The apoptosis index (AI) of HBECs was tested by flow cytometry. Levels of apoptosis-related protein were determined by Western blotting.

**RESULTS:**

PFD treatment significantly decreased the AI value in emphysema mouse lung tissue by TUNEL. In HBECs, flow cytometry showed that PFD could significantly reduce AI led by CSE. Both *in vitro* and *in vivo*, protein levels of Bax and Cleaved-caspase 3 in CSE group significantly increased in contrast with the control group; while Bcl-2 protein level in CSE group was significantly decreased; moreover, PFD significantly reversed protein level changes of Bcl-2, Bax, and Cleaved-caspase 3 led by CSE.

**CONCLUSIONS:**

This study reveals that PFD may potentially protect against CSE induced apoptosis.

## INTRODUCTION

As a global disease burden, chronic obstructive pulmonary disease (COPD) has ranked the third among all death causes worldwide^1^. Cigarette smoke is considered as the leading risk factor for COPD^2^. Apoptosis derived from lung structural cells is a significant upstream event involved in COPD pathogenesis^3^. Increased apoptosis of airway epithelial and T-cell is observed in COPD patients, and the increase remains despite smoking cessation^4^. Furthermore, inhibitors targeting cigarette smoke induced apoptosis are gradually investigated, such as melatonin^5^ and N-acetylcysteine^6^.

Pirfenidone (PFD) is an anti-fibrosis agent with acceptable side-effect profile, which has been proved by phase III clinical trial^7^. Besides the anti-fibrotic effect, PFD also has pharmacological functions including antioxidant and anti-inflammatory effects^8^. A recent study demonstrated that PFD protected cardiomyocytes against homocysteine induced apoptosis^9^. In addition, pirfenidone blocked apoptosis of lipopolysaccharide-induced lung alveolar epithelial type II cells^10^. However, there is no relevant study investigating the effect of PFD on cigarette smoke extract (CSE) induced apoptosis.

According to above findings, the current study was designed to explore whether PFD was able to mediate CSE induced apoptosis.

## METHODS

### CSE preparation

Following Chen et al.^11^, the solution of CSE was prepared freshly each time. Firstly, 20 mL serum-free cell culture medium or phosphate buffered saline (PBS) was added to a modified syringe-driven apparatus. Then, cigarettes (Furong, Hunan) were burned (10 cigarettes in animal experiments, and one cigarette in cell experiments), and the cigarette smoke was bubbled into the modified syringe-driven apparatus with a vacuum pump at constant pressure. Cigarette smoke and PBS (or serum-free cell culture medium) were fully mixed to get 100% CSE solution. Lastly, the microfilter (pore size: 0.2 μM) was used to filter CSE solution.

### Animal experiment

BALB/C mice, six weeks old, were divided into four groups randomly into: control group (n=6), emphysema group (n=6), 50 mg/kg/d PFD + emphysema group (n=6), and 100 mg/kg/d PFD + emphysema group (n=6). In order to establish emphysema model, a volume of 0.3 mL CSE solution was intraperitoneally injected to mice on days 0, 11, and 22, while 0.3 mL PBS were intraperitoneally injected to mice in the control group on days 0, 11, and 22. PFD (Beijing Kangdini Pharmaceutical Co. Ltd, Beijing, China) was intragastrically administered to mice in 50 mg/kg/d PFD + emphysema group and 100 mg/kg/d PFD + emphysema group for 28 days consecutively, while mice in the control group and emphysema group were intragastrically administered with equal volumes of normal saline daily. On day 29, mice were anesthetized and sacrificed after injecting pentobarbital^12^. Animal experiments were performed in the Animal Center of Hunan Provincial People’s Hospital, and the animal protocol was approved by Animal Care and Use Ethnics Committee of Hunan Provincial People’s Hospital.

### TUNEL analysis

The cell apoptosis in lung tissue was measured by a terminal deoxynucleotidyl transferase (TdT)-mediated dUTP nick end-labeling (TUNEL) assay (Hoffman-La Roche Ltd., Basel, Switzerland) following the instructions. Apoptotic index (AI) in the slides were observed and calculated under a morphometric microscope. Of note, fields containing non-parenchymal structures such as large airways or vessels were excluded^11^.

### Cell culture and treatment

Human bronchial epithelial cells (HBECs) were cultured in DMEM (Hyclone, Logan, UT, USA) added with 10% fetal bovine serum (Gibco, USA), 100 U/mL penicillin and 100 U/mL streptomycin (Thermo Fisher Scientific, Waltham, MA, USA) at 37°C in a 5% CO_2_ humidified incubator. To measure potential cytotoxicity of PFD on HBECs, cell viability of HBECs was examined after different concentrations (0, 250, 500, 750, 1000, and 1500 mg/L) of PFD exposure. To determine optimized CSE exposure concentration *in vitro*, AI of HBECs was measured by flow cytometry (FCM) analysis after exposing to different concentrations (0, 1, 5, and 10%) of CSE.

### Cell viability assay

Following the instructions, Cell Counting Kit-8 (CCK-8) assay kit (TargetMOI, USA) was applied to measure cell viability of HBECs. In brief, PFD was treated to HBECs (0, 100, 250, 500, 750, and 1000 mg/L) for 24 hours firstly in 96-well plate. Secondly, each well in 96-well plate was added with 10 µL CCK-8 testing solution. Thirdly, cells in 96-well plate were cultured with 5% CO_2_ in a humidified incubator at 37°C for two hours. Finally, the absorbance at 450 nm was read by a microplate reader and cell viability was further calculated.

### FCM analysis

HBECs collected from 6-well plates were washed with PBS twice, centrifuged (2000 rpm for 5 min), and added with 500 µL binding buffer. Then, 5 µL Annexin V-APC and 5 µL Propidium Iodide (PI) were mixed with resuspended cells, according to the instructions. Finally, AI of HBECs was examined by flow cytometer^11^.

### Western blotting analysis

Total protein extracted from lung tissue and HBECs was separated by sulfate polyacrylamide gel electrophoresis (SDS-PAGE) and then transferred to polyvinylidene fluoride (PVDF) membranes. A 5% non-fat milk in Tris-buffered Saline with Tween (TBST) was then applied to block PVDF membranes for one hour at room temperature. Primary antibodies against B-cell lymphoma-2 (Bcl-2) (Cell Signaling Technology, USA, 1:1000), Bcl-2 Associated X (BAX) (Abcam, USA, 1:1000), Cleaved-caspase 3 (Cell Signaling Technology, USA, 1:1000), and glyceraldehyde-3-phosphate dehydrogenase (GAPDH) (Abcam, USA, 1:1000) were incubated with PVDF membranes overnight at 4°C. Then HRP-labeled IgG secondary antibodies (Proteintech, Wuhan, China, 1:5000) were incubated with PVDF members for one hour at room temperature. For protein band densities, ImageJ software was applied to analyze quantitatively.

### Statistical analysis

Data in this study were analyzed by GraphPad Prism (GraphPad Prism 7.04, San Diego, CA, USA). The results are presented as mean ± standard error (SEM). One-way analysis of variance (ANOVA) combined with Tukey’s *post hoc* test was applied to conduct statistical comparisons. A p<0.05 was considered statistically significant.

## RESULTS

### Effect of PFD on cell apoptosis in CSE-induced emphysema mice

**Figure 1:**
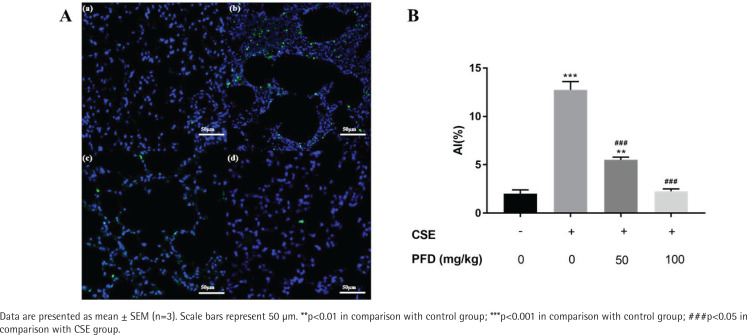
Effect of PFD on cell apoptosis in CSE-induced emphysema mice using TUNEL. A. TUNEL staining in mouse lung tissue: (a) Control group; (b) emphysema group; (c) 50 mg/kg/d PFD + emphysema group; (d) 100 mg/kg/d PFD + emphysema group. B. Statistical analysis of the AI in different groups

**Figure 2:**
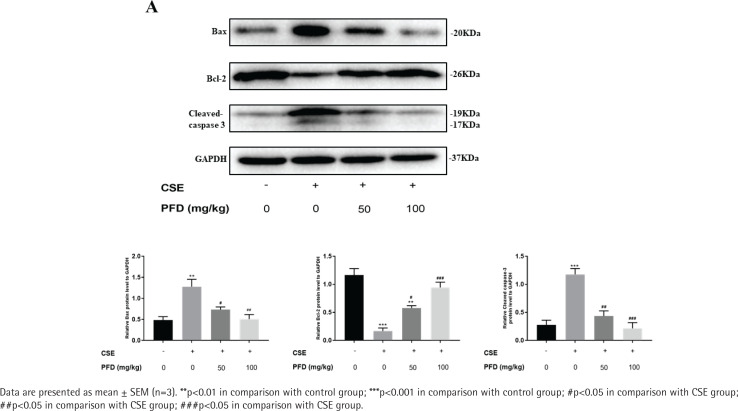
Effect of PFD on CSE-induced apoptotic protein expression in mouse lung tissue. A. Western blotting analyses of Bcl-2, Bax, and Cleaved-caspase 3 protein levels in mouse lung tissue. B. Statistical analysis of relative protein expression for Bcl-2, Bax, and Cleaved-caspase 3

In the CSE group, the alveolar space was significantly increased and the alveolar wall was dramatically destroyed and demonstrated alveolar fusion. These lung pathology changes give direct evidence of emphysema model. TUNEL assays demonstrated that AI of CSE group was significantly increased in contrast with the control group; PFD treatment significantly decreased AI compared with the CSE group. (Figures 1 A and B). Figure 2 shows protein levels of Bcl-2, Bax, and Cleaved-caspase 3 in mouse lung tissue. Compared with the control group, Bax and Cleaved-caspase 3 protein levels in the CSE group were significantly elevated; while Bcl-2 protein level in CSE group was significantly decreased. Moreover, PFD significantly reversed level changes of Bcl-2, Bax, and Cleaved-caspase 3 induced by CSE.

### Effect of PFD with different concentrations on cell viability of HBECs

**Figure 3:**
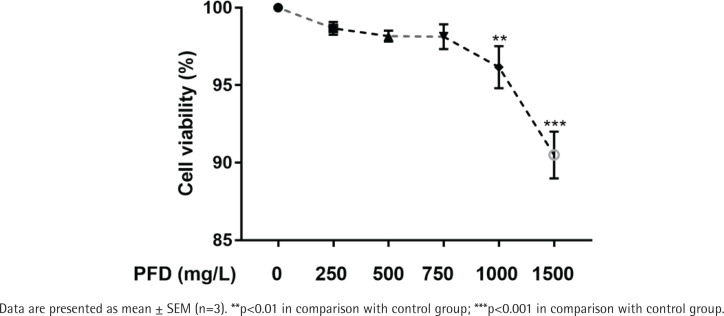
Cell viability of HBECs incubated with different concentrations of pirfenidone by CCK-8 assays

Cell viability of HBECs exposed to different concentrations of PFD is presented in Figure 3. When HBECs exposed to 0, 250, 500, and 750 mg/L PFD, there was no significant difference in cell viability in comparison with the control group. Nevertheless, exposure to PFD with 1000, 1500 mg/L for PFD significantly decreased cell viability of HBECs compared with the control group. And 750 mg/L PFD was selected for the treating concentration of HBECs in further studies.

### Effect of PFD on CSE induced apoptosis in HBECs

**Figure 4:**
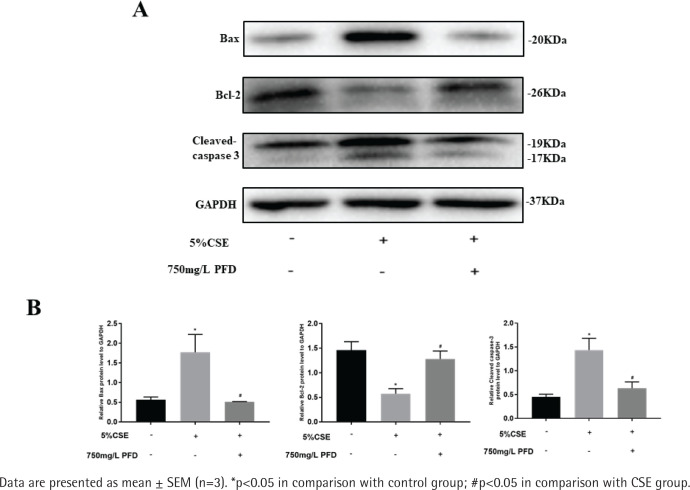
Effect of PFD on CSE-induced apoptotic protein expression in HBECs. A. Western blotting analyses of Bcl-2, Bax, and Cleaved-caspase 3 protein levels in mouse lung tissue. B. Statistical analysis of relative protein expression for Bcl-2, Bax, and Cleaved-caspase 3

Supplementary file Figure 1 presents different concentrations of CSE on cell apoptosis in HBECs. HBECs were exposed to CSE with different concentrations for 24 h and AI values were further measured by flow cytometry in each group. Compared with the control group, the values of AI were significantly increased in the 5% CSE group and 10% CSE group, while no significant increase in AI was found in the 1% CSE group. As shown in Supplementary file Figure 2, 750 mg/L PFD could significantly reduce the value of AI induced by 5% CSE. Western blotting analysis of Bcl-2, Bax, and Cleaved-caspase 3 in HBECs is presented in Figure 4. In CSE group and CSE+PFD group, HBECs were exposed to 5% CSE for 24 h. Protein levels of Bax and Cleaved-caspase 3 in 5% CSE group were significantly increased when comparing with the control group; while the protein level of Bcl-2 in 5% CSE group was dramatically decreased; interestingly, 750 mg/L PFD could significantly reverse protein changes of Bcl-2, Bax, and Cleaved-caspase 3 induced by CSE.

## DISCUSSION

Results from this study show that PFD significantly decreases the ratio of cell apoptosis induced by CSE in emphysema model and HBECs. Both *in vitro* and *in vivo*, PFD significantly reversed protein level changes of Bcl-2, Bax, and Cleaved-caspase 3 led by CSE. The results suggest that PFD potentially protects against CSE induced apoptosis.

In epithelial and endothelial lung cells, neutrophils, lymphocytes, and myocytes of smokers’ and in emphysematous lungs, abnormal apoptotic events have been observed^13^. Furthermore, gene expression analysis indicated that apoptotic genes of lung cells were related to cigarette smoke-induced emphysema in mice^14^. It was found that apoptosis of HBECs was increased in COPD^15,16^. In this study, apoptosis index in CSE-treated HBECs and emphysema model was significantly elevated, which was consistent with previous studies. Kuo et al.^17^ proved that cigarette smoke-induced apoptosis might be stimulated by the stabilization of p53, resulting in an increase in the ratio of Bax/Bcl-2 and activation of caspase cascade. We also found cigarette smoke could induce an increase in Bax and Cleaved-caspase 3, while the protein level of Bcl-2 was dramatically decreased after CSE treatment.

Potential anti-apoptotic effects of PFD have been reported in previous studies^9,10,18-20^. Chen et al^18^. found that PFD could inhibit in tubular cell apoptosis by maintaining mitochondrial membrane stability, and subsequently inhibit the mitochondrial apoptotic signaling pathway. It was also observed that PFD was able to reverse pro-apoptotic properties of chronic cyclosporine A (CsA) in a nephrotoxicity animal model, and this may result from decreasing p53 and Fas-L expression, and increasing survival gene Bcl-xL expression^19^. Tsuchiya et al.^20^ reported that PFD treatment was capable of reducing LPS induced liver injury and hepatic apoptosis by inhibiting NF-κB pathway.

## CONCLUSIONS

Our *in vivo* and *in vitro* studies demonstrate that cell apoptosis is involved in COPD pathogenesis. Our results suggested the potential role of PFD protecting apoptosis induced by CSE, which may provide a treatment alternative for COPD.

## Data Availability

The data supporting this research are available from the authors on reasonable request.
