# Supplementary material for: Effect of pirfenidone protecting against cigarette smoke extract induced apoptosis
Source: Tob Induc Dis. 2022 Mar 1;20:24. doi: 10.18332/tid/146169 (PMC8886422; doi:10.18332/tid/146169)
Supplement: Supplementary file 1 [file TID-20-24-s1.pdf]

Supplementary file

Supplemental Figure 1. Effect of varying concentrations of CSE-induced apoptosis in HBECs. A. Measurement of apoptosis using Annexin V-APC/PI staining and FCM; B. Statistical analysis of the AI in different groups. Data are presented as the mean ± SEM (n=3). *** p<0.001 in comparison with control group.

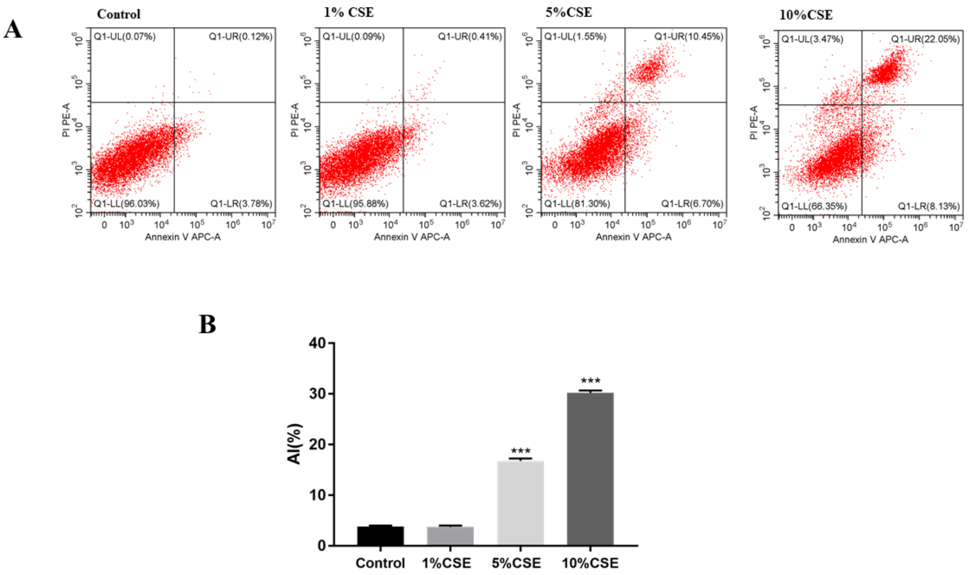

Supplemental Figure 2. Effect of PFD on CSE-induced apoptosis in HBECs. A. Measurement of apoptosis using Annexin V-APC/PI staining and FCM; B. Statistical analysis of the AI in different groups. Data are presented as the mean ± SEM (n=3). *** p<0.001 in comparison with control group; ### p<0.05 in comparison with CSE group.

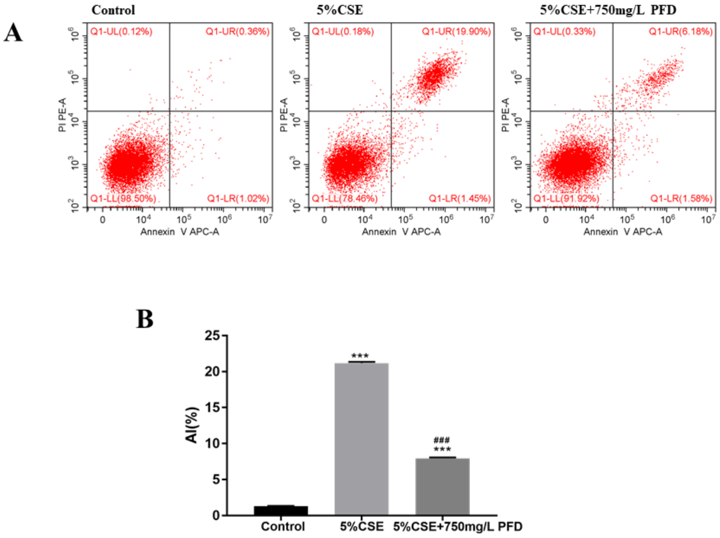

© 2022 Ma Y. et al.
